# Roles of Noncoding RNAs in Ciliate Genome Architecture

**DOI:** 10.1016/j.jmb.2019.12.042

**Published:** 2020-07-10

**Authors:** Sarah E. Allen, Mariusz Nowacki

**Affiliations:** Institute of Cell Biology, University of Bern, Switzerland

**Keywords:** ciliates, noncoding RNA, genome rearrangement, transposon defence

## Abstract

Ciliates are an interesting model system for investigating diverse functions of noncoding RNAs, especially in genome defence pathways. During sexual development, the ciliate somatic genome undergoes massive rearrangement and reduction through removal of transposable elements and other repetitive DNA. This is guided by a multitude of noncoding RNAs of different sizes and functions, the extent of which is only recently becoming clear. The genome rearrangement pathways evolved as a defence against parasitic DNA, but interestingly also use the transposable elements and transposases to execute their own removal. Thus, ciliates are also a good model for the coevolution of host and transposable element, and the mutual dependence between the two. In this review, we summarise the genome rearrangement pathways in three diverse species of ciliate, with focus on recent discoveries and the roles of noncoding RNAs.

## Highlights

• Ciliate genomes undergo massive rearrangement and reduction during development.
• Transposon elimination is guided by small RNAs and carried out by transposases.
• New pathways for noncoding RNA production have recently been discovered in ciliates.
• Diverse ciliate species have different mechanisms for RNA-guided genome remodeling.

## Introduction

An example of the diversity of the functions of noncoding RNAs across evolutionary distance is their role in the fascinating genome rearrangement processes that take place in different species of ciliated protozoa (ciliate). In the last two decades, noncoding RNAs have been shown to have crucial roles in these processes, roles that are divergent both within and across species and processes. Genome rearrangement is a process fundamental to the development of many ciliates and is thought to have emerged around one billion years ago, before the divergence of spirotrichea and oligohymenophorea, as a defence against transposable element invasion of the genome [1,2].

### Nuclear dimorphism

One of the hallmarks of ciliates is their nuclear dimorphism: the presence of two distinct nuclei within the same unicellular organism. The larger nucleus is termed the macronucleus and contains all the DNAs required for the day-to-day life of the ciliate, often at a high copy number (up to 2000n for certain nanochromosomes in *Oxytricha*), due to the large complex cells. The smaller nucleus (termed the micronucleus) comprises the germline and is kept mostly silent and quiescent until it is required to build a new soma via sexual reproduction. Ciliates reproduce sexually through conjugation, in which two ciliates of opposite mating types join and exchange one pronucleus produced from meiotic and mitotic divisions of the germline micronucleus. This haploid pronucleus fuses with a second pronucleus in the recipient cell and undergoes a number of mitotic divisions, the products of which go on to form the new micronuclei and macronuclei (the numbers of the various nuclei vary with the species of ciliate). The old macronucleus is degraded and a new one is formed from one of these nuclei. Thus, like in other organisms, the germline is immortal whereas the soma dies and is replaced every generation. In *Paramecium*, sexual development can also occur without a partner, in a process known as autogamy. Here, the two haploid pronuclei produced in a developing cell simply fuse with each other, and development continues similarly to in a conjugating cell.

The ciliate germline can be compared with the germlines of more well-known organisms in that it must remain totipotent, with the ability to differentiate into any type of somatic nucleus, but must also manage the risks inherent to totipotency, namely transposon replication and invasion. A ciliate micronucleus contains a large proportion of transposable elements (TEs) [3], many of which may still be active and could replicate and reinsert themselves if not strictly controlled. The way that the micronucleus prevents TE replication is to remain almost exclusively silent, with no detectable transcription taking place during the vegetative (growth) phase of the life cycle [4]. The macronucleus must, of course, be transcriptionally active and is protected from transposon invasion through an unusual mechanism – all the transposons and transposon-derived sequences are removed by excision during the development of the macronucleus from the micronucleus. This produces a streamlined somatic genome consisting almost entirely of genes and gene regulatory elements.

### Genome rearrangement

The development of a new macronucleus is a staggeringly complex feat of DNA excision and repair. Apart from transposable elements, microsatellites, and other and repetitive DNA, ciliate germlines also contain large numbers of noncoding sequences termed internal eliminated sequences (IESs), which are remnants of ancient TEs and which frequently interrupt coding genes. In *Tetrahymena*, 34% of the micronuclear genome is removed in around 12,000 sections, whereas at the same time, the chromosomes are fragmented via de novo telomere addition and amplified to produce ~200 macronuclear chromosomes, at a ploidy of 45n, from the five diploid chromosomes present in the micronucleus [[5], [6], [7]]. This all happens accurately and reproducibly over a period of 12 h. In *Paramecium*, the same process removes 25% of the germline genome in over 45,000 fragments, whereas amplifying the ploidy to ~800n [8]. Perhaps the most impressive genome rearrangement occurs in the spirotrichea such as *Oxytricha*, in which 95% of the micronuclear genome is removed and the remaining 5% is reassembled into 16,000 tiny nanochromosomes, most of which contain a single gene each. In addition, around 20% of *Oxytricha* genes are scrambled in the micronucleus, meaning that they exist in several fragments that may not be adjacent or even in the correct order, and must be rearranged to form functional genes in the new macronucleus [9]. The sheer scale and complexity of the excision and repair that must occur during macronuclear development in ciliates is quite remarkable, and the questions of how the cell can identify which sequences to retain, the precise breakpoints, which broken ends to reanneal, etc., is the subject of much study.

### RNA-guided genome rearrangements

It has long been known that genome rearrangement configurations are inherited maternally, through a cytoplasmic factor that guides the removal of TEs and IESs based on their presence or absence in the parental macronucleus. This was first demonstrated with crosses performed with the d48 strain, a strain of *Paramecium* in which the germline is wild-type but the somatic macronucleus harbours a deletion in a region containing the surface antigen A gene. The cells were shown to transmit the deletion maternally through an unknown cytoplasmic factor [10,11]. Later, it was shown in both *Paramecium* and *Tetrahymena* that excision of germline-limited sequences could be prevented in the new macronucleus via microinjection of their retained forms into the maternal macronucleus, demonstrating that the maternal inheritance is sequence-dependent [[12], [13], [14]]. In the beginning of the 21st century, it was demonstrated that noncoding RNA is the factor that mediates this cytoplasmic inheritance [[15], [16], [17]]. Since then, a range of different classes and functions of RNAs have been discovered and their functions in the genome rearrangement process elucidated.

Ciliate noncoding RNAs have pivotal roles in determining which micronuclear sequences to retain in the new macronucleus and in which order. Noncoding RNAs are thus used as a means of information transfer from the old macronucleus to the new developing one, directing the development of the daughter macronucleus to mimic the maternal macronucleus. In other words, information about the state of the maternal soma is passed to the daughter soma without affecting the germline, allowing true transgenerational epigenetic inheritance while maintaining the Weismann barrier. In this review, we will discuss the various roles of noncoding RNA in genome rearrangements in ciliates, with focus on recent developments in the three most-studied organisms *Paramecium*, *Tetrahymena*, and *Oxytricha*.

## IESs, background

The genome rearrangement pathways in ciliates provide an interesting example of the coevolution of parasitic DNA and its host, and their interdependence. While the IES excision pathways evolved as a defence against TEs, they also heavily rely on TE and TE-derived sequences for their function. The clearest example of this is the excisases that perform the excision of IESs, they are transposases that have been co-opted by the cell to remove transposons and transposon-derived sequences. The requirements of the transposases impose limitations on the TEs and IESs that can remain in the germline – if an IES cannot be removed during development then it will not persist over evolutionary time. Therefore, IESs in different ciliates have different distinctive characteristics, reflecting the requirements of the excision machineries. The transposases and their requirements for excision will be discussed later. As IESs are the remnants of ancient TEs and the mechanisms for their removal are largely the same, for simplicity we will henceforth refer to all sequences that are excised and removed from the germline during ciliate development as IESs.

### IESs characteristics

In *Tetrahymena*, there are around 12,000 IESs that must be removed during development. They are highly repetitive, with plenty of sequence overlap between different IESs, and range in size from 134 bp to 43.4 kbp (with 85% of IESs between 1 and 10 kbp). The IESs are largely situated in subtelomeric and centromeric gene-poor regions, and therefore do not interrupt coding sequences [7]. There are also a number of chromosomes that form transiently during sexual development but are not maintained in the vegetative life cycle, these are termed nonmaintained chromosomes (NMCs) and contain actively transcribed genes that are important during development [7,18]. In *Tetrahymena*, IES removal is imprecise, with microheterogeneity and sometimes large variability observed in IES borders [6,7,18]. This is tolerated by the cells due to the absence of IESs in coding regions. However, recently a small class of 12 IESs situated within coding genes was discovered, the excision of which is highly precise so as not to interrupt the coding sequence [19].

In *Paramecium*, in the range of 45,000 IESs are removed from the germline during development. These are much shorter than the *Tetrahymena* IESs, ranging from 26 bp to 5 kbp with a median length of 51 bp and a mode (most frequently found length) of 28 bp [8,20]. 93% of IESs in *Paramecium* are under 150 bp long and over a third are between 26 and 31 bp. *Paramecium* IESs are spread all over the genome, including within coding sequences, meaning that their excision has to be extremely precise so as not to interrupt the reading frame of the genes they inhabit. They are flanked by TA dinucleotide repeats, one of which is removed along with the IES and one of which is retained in the macronuclear sequence. They also exhibit a loose end consensus inverted repeat (IR) sequence of TAYAG, which varies slightly with the size of the IES and its dependence on small RNAs for excision (see section on piwi-associated RNAs) [20,21]. Interestingly, *Paramecium* IESs show a sinusoidal length distribution which peaks at 28 bp and then has progressively smaller peaks every ~10.2 bp, except for 38 bp where hardly any IESs are found. Although it is not clear exactly what causes this length distribution, it is hypothesised that it has something to do with bending of the DNA during the excision process, as 10.2 bp corresponds to one turn of the double helix in B-form DNA [20,22].

*Oxytricha* IESs make up 95% of the germline genome and the macronuclear-destined sequences (MDSs) are frequently scrambled and reversed. Owing to this scrambling, the smallest *Oxytricha* IES is 0 bp, as it is simply two adjacent scrambled MDSs. The sizes of *Oxytricha* IESs vary with whether the MDSs they flank are scrambled or not, scrambled IESs are shorter (median 27 bp) than nonscrambled IESs (median 61 bp). MDSs align by way of flanking “pointer” sequences, which are between 2 and 20 nt long [3,23,24]. The pointers behave similarly to the TA repeats in *Paramecium* in that they are located on either end of an MDS, and one pointer is retained in the macronuclear genome after rearrangement while one is removed along with the IES. Like *Tetrahymena*, *Oxytricha* has a number of nonmaintained chromosomes that are formed and then removed during sexual development. These NMCs express 810 protein-coding genes that are almost exclusively conjugation-specific [3].

## Piwi-associated small RNAs in genome rearrangements

PIWI-associated RNAs, or piRNAs, are a well-studied class of small RNAs found in metazoan germlines and necessary for the protection against TE expansion [25]. In some ciliates, Piwi-bound small RNAs help to direct the elimination of germline-limited sequences including transposable elements, minisatellites and IESs during the development of the new macronucleus [15,26]. In this sense, they can be seen as analogous to the piRNAs in germlines of animals, only the latter direct transcriptional silencing and heterochromatinisation of TEs while the former direct their complete removal from the genome. Interestingly, *Oxytricha* piRNAs act in the opposite way to the scnRNAs of *Paramecium* and *Tetrahymena*, targeting sequences for retention rather than elimination. In this section, the IES-targeting small RNAs of *Paramecium* and *Tetrahymena* will be discussed and compared in detail first, and *Oxytricha* piRNAs will be addressed later.

### scanRNAs

**Fig. 1:**
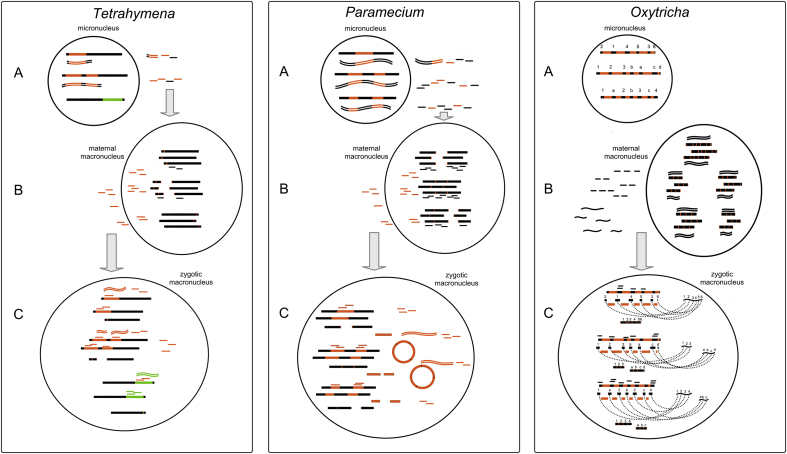
The RNA-guided genome rearrangements of three ciliate species. Thick horizontal lines depict chromosomes; IESs are orange, MDSs are black. Long noncoding RNA is represented by wavy lines, small Piwi-bound RNAs are depicted as short horizontal lines. Proteins are mentioned in the legend but not depicted for simplicity. Left, *Tetrahymena*. A: dsRNA scnRNA precursors are transcribed bidirectionally from IES-rich A-regions in the meiotic micronucleus. They are cleaved by a Dicer-like enzyme into short, 26–32 nt long early-scnRNAs which are imported into the maternal macronucleus. B: Here, scnRNAs that match to macronuclear sequence are removed and the remaining IES-matching scnRNAs are transported to the developing zygotic macronucleus. C: In the developing macronucleus, while amplification and chromosome fragmentation are ongoing, scnRNAs target both type A (orange) and type B (green) IESs for heterochromatinisation and elimination, which triggers transcription of late-scnRNAs from both type A and type B IESs. Late-scnRNAs then further target the amplifying copies of IESs to ensure complete elimination. Middle, *Paramecium*: A: scnRNA precursors are transcribed uniformly from the micronuclear genome and cleaved by Dicer-like enzymes Dcl2 and Dcl3 to 25 nt scnRNAs. The scnRNAs are transported by Piwi proteins into the maternal macronucleus, B, where scnRNAs that find matches are removed. The remaining IES-matching sequences are transported to the developing zygotic macronucleus, C. Here they target IESs for excision. Once excised, IESs concatenate end on end to form circles, which are transcribed bidirectionally and cleaved by a second Dicer-like enzyme, Dcl5, to form iesRNAs. iesRNAs then target amplifying copies of IESs to ensure complete excision. Meanwhile, chromosomes are fragmented and telomerised. Right, *Oxytricha*: A: The *Oxytricha* macronucleus contains a huge number of IESs and its genes are scrambled, depicted as numbered/lettered gene fragments out of order. B: Both piRNA precursors and guide RNAs are produced from bidirectional transcription of the short nanochromosomes in the maternal macronucleus. They are transported to the zygotic macronucleus where the piRNAs target macronuclear-destined sequences for retention, and IESs are removed. The guide RNAs help to arrange the macronuclear-destined sequences in the correct order on gene-sized chromosomes.

The scanning model for IES and TE elimination in *Paramecium* and *Tetrahymena* was first proposed in 2002 in *Tetrahymena* [15], and elegantly explains how information about which micronuclear sequences are present in the old maternal macronucleus is transmitted to the developing macronucleus. In the early stages of conjugation, the entire micronucleus is bidirectionally transcribed and cleaved to form small RNAs, termed scanRNAs or scnRNAs. The scnRNAs are bound to PIWI proteins and are transported to the maternal macronucleus, where they are compared against the entire macronuclear genome. The scnRNAs that find a perfect match are removed and degraded. The scnRNAs that fail to find a match, by definition, correspond to sequences not present in the maternal macronucleus and therefore not desired in the new macronucleus. These scnRNAs are sent to the developing macronucleus where they target their matching sequences for elimination [15,16,27,28]. The scnRNA pathway is outlined in Fig. 1, panels 1 and 2 (*Tetrahymena* and *Paramecium*), with the similarities and differences outlined.

### scnRNA production

More is known about scnRNA production in *Tetrahymena* than in *Paramecium*. In *Tetrahymena*, the premeiotic micronuclear genome is bidirectionally transcribed by RNA polymerase II (RNApolII) [[11], [13], [14], [15], [29]], and then cleaved by a Dicer-like enzyme, Dcl1, into 26–32 nt long RNAs [30,31]. These are then loaded onto the Piwi protein Twi1, 2′ O-methylated, and transported to the maternal macronucleus [15,27,32]. On loading of the scnRNA, the passenger strand is cleaved by the Slicer domain of Twi1. The passenger strand removal is necessary both for the stable accumulation of scnRNAs and for the transport of the Twi1-scnRNA complex to the parental macronucleus. The latter is mediated by a protein called Giw1, which binds to the mature Twi1-scnRNA complex and escorts it to the nucleus [33]. scnRNAs in *Tetrahymena* have certain sequence features including a strong 5′ U bias and a weaker A bias 3 bases from the 3′ end. These base preferences are indicative of Dicer cleavage, which cuts dsRNA with a 2 nt 3′ overhang and a preference for 5' U [34]. The transcription of scnRNA precursors is a well-coordinated process that requires the rapid global activation of a normally completely silent nucleus and occurs shortly before chromosome condensation in meiosis I [35]. Recently, developmental-specific components of the transcriptional regulator complex Mediator have been characterised in *Tetrahymena* and shown to localise to the premeiotic micronucleus where they coordinate the burst of transcription associated with scnRNA production [36]. In *Tetrahymena*, transcription of the micronucleus is not uniform as initially expected, rather it is biased towards repetitive regions and a class of IESs called type A IESs [21,22]. This preferential transcription of non–macronuclear-destined sequence appears to be directed by the mediator-associated protein Rib1, which directs RNApolII to pericentromeric and subtelomeric regions where the repetitive regions are located [36].

In *Paramecium*, the transcription of the micronucleus has not been globally measured, so it is not clear whether transcription is uniform or whether there is a bias towards non–macronuclear-destined sequences. However, it is assumed that the transcription is uniform based on the relative abundances of IES-matching and MDS (macronuclear-destined sequence)-matching scnRNAs during early development, along with the fact that in *Paramecium*, IESs and transposable elements are spread much more uniformly across the genome than in *Tetrahymena* [20,39]. The cleavage of scnRNA precursors in *Paramecium* is carried out by two Dicer-like enzymes, Dcl2 and 3, which have complementary functions in producing scnRNAs. Dcl2 is responsible for the scnRNA length, precisely 25 nt, whereas Dcl3 conveys a sequence cleavage preference of 5′UNG [39,40]. After their production, *Paramecium* scnRNAs are loaded onto two Piwi proteins, Ptiwi01 and Ptiwi09, which have 98% identity at the amino acid level and are thought to have identical functions [26,41].

## scnRNA selection

The Piwi-complexed scnRNAs are transported to the maternal macronucleus where the genomewide comparison event called “scanning” takes place [27,28,39]. During scanning, the scnRNA pool is enriched in IES- and TE-matching scnRNAs, presumed due to a loss of MDS-matching scnRNAs (although an amplification of IES- and TE-matching scnRNAs cannot be ruled out) [38,39]. The details of how this “scanning” takes place are still mysterious, although it appears that the PIWI-complexed scnRNAs bind to genomewide transcripts rather than to the genomic DNA itself. This notion is based on experiments in *Tetrahymena*, where the RNA helicase Ema1p has been shown to be necessary for binding of scnRNA-Twi1 to macronuclear chromatin, and nascent transcripts from the parental macronucleus were detected by RT-PCR [42]. Such transcripts have also been detected in *Paramecium* and are necessary for the maternal inheritance of excision and retention of certain IESs [28]. Whatever the precise mechanism for scnRNA selection in the old macronucleus, it must be agreed that it is stunningly complicated process, the logistics of which are almost unimaginable. Take *Paramecium*, arguably the simplest genome rearrangement system with only ~25% of its 98 Mb germline sequences removed during development. If the entire micronuclear sequence is bidirectionally transcribed only once, it would give rise to 2*98Mb/25 nt = 3.92 million unique scnRNAs, each of which must scan 72 Mb worth of macronuclear genome sequence to find its perfect match. Unless there is an unknown mechanism helping to guide scnRNAs to their matching macronuclear sequences, the number of scnRNA-genome interactions required is astronomical. This entire process takes place in a period of 2–4 h.

## scnRNA targeting of DNA for elimination

The scnRNAs remaining after scanning has taken place are those mapping to regions destined for elimination in the new macronucleus. Still in complex with their Piwi proteins, they are transported to the developing macronucleus where they again perform a genomewide scanning event, this time to find matching sequences to target for elimination. In *Tetrahymena*, the targeting is fairly well understood and involves heterochromatinisation via methylation of histone H3 lysine 9 and 27 (H3K9 and H3K27) [[43], [44], [45]]. This is dependent on the scnRNA-Twi1 complex and on the histone methyltransferase Ezl1, a homologue of *Drosophila* E(z). The methylation of H3K9 and H3K27 leads to binding and heterochromatin assembly by the HP1 homologue Pdd1. Pdd1 is likely the effector molecule for heterochromatinisation, recruiting a number of downstream factors including the excision machinery which removes the heterochromatinised DNA [46,47]. The heterochromatinised IESs and TEs group into so-called heterochromatin bodies or elimination bodies, which can be seen as distinct foci in the nucleus when staining for heterochromatin or one of the proteins involved in DNA elimination [48,49]. Heterochromatin body formation is dependent on the dephosphorylation of Pdd1, which is hyperphosphorylated on heterochromatin formation [50,51]. As yet, it is unclear whether the heterochromatin body formation occurs before or after excision of the IESs and TEs has begun; however, it seems to be necessary for excision as knockout of proteins involved in heterochromatin body formation leads to retention of IESs in the daughter macronucleus [[52], [53], [54], [55], [56]]. Interestingly, one of these proteins is an RNA-binding protein that forms prion-like aggregates, suggesting that RNA may be involved in the aggregation of heterochromatin into foci [56].

In *Paramecium*, the histone methyltransferase Ezl1 is also active during sexual development and is necessary for the deposition of H3K9me3 and H3K27me3 marks in new macronuclei [57,58]. In the developing macronucleus, H3K9me3 and H3K27me3 form foci similarly to *Tetrahymena*; these foci progressively become smaller and fewer in number as development progresses. Recently, H3K9 and H3K27 marks have been shown to be present on a number of transposable elements [58]. However, unlike in *Tetrahymena*, in *Paramecium,* the heterochromatin marks have not been shown to directly bind the excision machinery, and so indirect effects such as effects on the expression of relevant genes cannot be ruled out. Notably, in *Paramecium,* most IESs are significantly shorter than the binding footprint of a nucleosome, and many of these short IESs are dependent on Ezl1 for their excision [57]. How histone marks could be responsible for targeting these IESs for elimination is not understood. A chromatin assembly factor called PtCAF-1 is also necessary for H3K9me3 and HeK27me3 deposition in the zygotic macronucleus and has a similar localisation pattern to Ezl1, suggesting that they may interact. Interestingly, PtCAF-1 appears to be necessary for the completion of scanning in the maternal macronucleus; when it is removed the MDS-matching scnRNAs are not eliminated [59].

### Late-scnRNAs and iesRNAs

During the TE and IES elimination process, the genomes of *Tetrahymena* and *Paramecium* are amplified up to 45n and 800n, respectively. In both species, a second class of IES-targeting small RNAs has evolved that is active during the DNA amplification stage of new macronuclear development [37,39]. It is hypothesised that this secondary small RNA pathway has evolved to ensure complete removal of all copies of IESs in a rapidly amplifying but only partially rearranged genome. In *Tetrahymena*, the secondary RNA pathway is named “Late-scnRNAs” due to the RNAs' production in the later part of the developmental process. They are produced in the zygotic macronucleus from all IESs and are around 29 nt in length [37]. Late-scnRNAs are thought to be produced by Dcl1 and they bind to Twi1; however, interestingly, they only bind to the zygotic Twi1 as opposed to early scnRNAs which bind maternal Twi1. The discovery of the late-scnRNAs led to the division of *Tetrahymena* IESs into two groups, termed type A IESs and type B IESs. Type A IESs produce early-scnRNAs, whereas type B IESs produce no scnRNAs in early development. In late development, both type A and type B IESs produce late-scnRNAs in an early-scnRNA–dependent manner, so if the early-scnRNA pathway is silenced then no late-scnRNAs are produced. A loss of late-scnRNAs leads to the failure to excise type B IESs. This can be explained by sequence overlap between the two types of IESs: a subset of scnRNAs from type A IESs will bind to type B IESs in *trans* and initiate heterochromatinisation, which leads to production of late-scnRNAs by an unknown mechanism. Late-scnRNA production has been shown to take place before IES excision and is not excision-dependent [60]. In some ways, the production of secondary scnRNAs through targeting of genomic regions by primary scnRNAs can be seen as analogous to the secondary piRNA production in flies and mice (for review see Ref. [61]), where transposon transcripts are targeted by primary piRNAs and cleaved to form secondary piRNAs. However, there are some key differences between the systems: for example, in ciliates, the Late-scnRNA precursors are transcribed only in response to targeting by early-scnRNAs, as opposed to being active transposon mRNA, and are cleaved by a Dicer-like enzyme rather than by the primary piRNA-bound Argonaute.

In *Paramecium*, the second class of small RNAs is named iesRNAs, as the RNAs exclusively bind to IESs and TEs. Their length varies from around 26–31 nt, and they have a weak end consensus sequence of 5′UAG. Like in *Tetrahymena*, iesRNAs are produced in the developing zygotic macronucleus and are necessary for the excision of a subset of IESs. In contrast to *Tetrahymena*, iesRNAs are produced from all IESs, are cleaved by a distinct Dicer-like enzyme, Dcl5, and bind to their own Piwi proteins, Ptiwi10 and Ptiwi11 [[39], [40], [41]]. Their production is rather interesting: following scnRNA targeting and excision of IESs, the excised IESs are ligated at their ends to provide a circular template for dsRNA transcription and cleavage by Dcl5. Most IESs are too short to circularise, these shorter IESs concatenate together end on end until they reach a length whereby they can circularise and provide a template for RNA polymerase [62]. Dcl5 is able to reliably produce iesRNAs that correspond to IESs from these randomly assembled templates thanks to its sequence cleavage preference, which recognises and cleaves at IES-IES junctions [40].

Through the secondary small RNA pathway, both *Tetrahymena* and *Paramecium* establish a positive feedback loop which leads to large numbers of secondary IES-targeting RNAs being produced and ensuring complete removal of all IES sequences in the highly polyploid zygotic macronucleus.

### piRNAs in *Oxytricha*

A class of small piwi-binding RNAs is produced during *Oxytricha* development and is necessary for correct development of the new macronucleus. In contrast to *Tetrahymena* and *Paramecium*, *Oxytricha* piRNAs are produced in the maternal macronucleus and identify sequences to be retained, rather than sequences to be excised. This makes sense given that the vast majority (~95%) of the germline is removed during macronuclear development, so identifying the relatively few macronuclear-destined sequences is energetically more efficient. *Oxytricha* piRNAs are 27 nt long and bind to the Piwi protein Otiwi1 [63,64]. How the piRNAs target sequences for retention is not known; however, a model based on recent work in the related ciliate *Stylonychia* proposes a mechanism based on replication stalling caused by binding of the Piwi-RNA complex. In this model, the bound Piwi-RNA complex prevents replication at macronuclear-destined sequences and thus inhibits replication-dependent H3K27 methylation, which in turn leads to sequence elimination [65].

## Non-Piwi RNAs in ciliate development

### Guide RNAs in *Oxytricha*

Owing to the scrambled nature of the *Oxytricha* micronuclear genome, identifying the sequences that are to be removed versus retained is not sufficient information to produce a functioning macronucleus. As described earlier, *Oxytricha* IESs and MDSs contain pointer sequences at their ends which help to identify adjacent MDSs [3,24]. However, the pointer sequences are not unique and in some cases are very short, meaning that they do not in themselves provide enough information to reliably unscramble the germline. To guide the rearrangement process, *Oxytricha* generates long RNAs from the parental macronucleus that therefore correspond to the rearranged genome. These “guide RNAs” are then transported to the new developing macronucleus where they are necessary for guiding the correct arrangement of MDSs [66,67]. Transcription of the guide RNAs appears to rely on a specific subunit of RNA polymerase II that arose from a gene duplication in stichotrich ancestors and has evolved to exclusively transcribe developmental guide RNAs [68]. It is possible to disrupt and alter the arrangement of the zygotic macronuclear genome through injection of alterative guide RNAs, demonstrating that changes in the parental macronucleus can be directly inherited via epigenetic processes. The experiments that showed this indicate that the guide RNAs act as a template or scaffold for host DNA rearrangement, rather than being involved in homologous recombination or similar. This is based on the observation that point substitutions included in injected alternative guide RNAs were generally not transmitted to the alternatively rearranged genomic DNA sequence [66].

### Other *Oxytricha* noncoding RNAs

Interestingly, it was recently shown that *Oxytricha* TEs and nonrepetitive micronucleus-limited sequences are circularised on excision, and transcribed to form noncoding RNAs, similarly to *Paramecium* iesRNA production. While it is not yet clear what the *Oxytricha* RNAs are for, both circularisation and transcription are performed in a nonrandom fashion and peak during middevelopment, suggesting that the RNAs may have a function in genome rearrangement [69].

Recently, a class of small RNAs in *Oxytricha* has been discovered that regulates DNA copy number during vegetative growth [70]. It was previously known that noncoding RNAs produced during conjugation guide copy number of nanochromosomes in the offspring [71]. Together, these studies illustrate the profound importance of noncoding RNAs for directing every level of genome architecture in *Oxytricha*.

## Excisases and their requirements

The main enzyme responsible for IES removal in *Tetrahymena* is the domesticated PiggyBac transposase TPB2, which removes the imprecisely excised most IESs [72,73]. TPB2 has been shown to bind heterochromatin; hence, its recruitment to heterochromatinised IESs [65], but how the IES boundaries are identified was until recently mysterious. It has been shown that IESs are excised as complete units and circularised, rather than being sliced up into small pieces [[74], [75], [76]]. Thus, a mechanism for identifying IES boundaries and directing TPB2 cleavage must exist. It was known that *cis*-acting sequences helped to guide the excision from experiments in which shifting of certain IES-flanking sequences led to shifting of excision boundaries, but these sequences varied from IES to IES and no common consensus could be found [73,[77], [78], [79]]. Recently, a genomewide approach to search for IES-flanking IR sequences was undertaken, and it was found that there are several conserved IR sequences that flank different subsets of IESs at similar distances on both sides of the IES [80]. The known IES boundary-defining protein LIA3 was found to aid in the excision of a subset of IESs, and these LIA3-dependent IESs share similar polypurine-rich IR sequences at their boundaries [80,81]. Meanwhile, a second boundary-defining protein, Ltl1, was found to be required for the excision of another subset of IESs. These Ltl1-dependent IESs also share an IR sequence at their boundaries that is distinct from the LIA3-dependent IESs' IR sequence [82]. Together, these studies suggest a model for IES boundary recognition that involves a number of boundary-defining proteins, each identifying a unique IR sequence flanking a subset of IESs. The boundary-defining proteins recruit or activate TPB2, allowing coordinated cleavage of each end of an IES.

In addition to TPB2, two more PiggyBac-like transposases necessary for genome rearrangement in *Tetrahymena* have been discovered. These are named TPB1 and TPB6 and together they are responsible for the excision of the 12 IESs located within protein-coding genes, the excision of which is by necessity highly precise [19,83]. Interestingly, TPB6 is found on one of the nonmaintained chromosomes that appear transiently during development. The TPB1/TPB6-dependent IESs are flanked by the IR sequence TTAACHCTW, the TTAA from which is retained in the macronuclear genome. This indicates that the excision is similar to a canonical PiggyBac transposase event, although the IR is not alone sufficient for TPB6- and TPB1-mediated excision [83].

The only required end sequence for *Paramecium* IESs is the flanking TA repeat; however, a loose IR consensus of TAYAG exists. This sequence is not necessarily identical at each end of the IES and many IESs exist with widely differing end sequences [8,20,21]. Recently, a protein required for excision of a subset of IESs, with specific end sequences, was discovered [84], raising the possibility that *Paramecium* may have a system similar to the Lia3-like proteins in *Tetrahymena*, whereby IESs depend on different proteins for excision depending on their specific end sequences. The enzyme that carries out the excision of IESs is another PiggyBac-like domesticated transposon called PiggyMac (PGM) [85]. PGM cleaves with a 4 nt 5' overhang centred over the flanking TA [86,87], and requires both ends of each IES to be recognised and bound by both PGM and the nonhomologous end-joining machinery before it can cleave [22,88]. How this remote communication between IES ends occurs is not entirely clear; however, it was recently shown that PGM does not perform the IES excision alone, rather it has a large number of “PiggyMac-Like” (PGML) cofactors that are necessary for the PGM-catalysed cleavage to occur [89,90]. One model for IES excision involves a huge complex of PGM and PGML proteins spanning both IES ends and thus coordinating cleavage [90].

It was thought that all *Paramecium* IESs are dependent on PGM for their excision; however, recent progress in assembling the germline genome has called this into question, as a number of TEs and other noncoding elements were discovered in the germline genome assembly that are not present in genomic DNA isolated from PGM-silenced cells [8]. If there are non–PGM-dependent IESs, their excisase has yet to be found. So far, no NMCs such as the one carrying TPB6 have been discovered in *Paramecium*; however, their existence is a possibility that cannot yet be ruled out.

In *Oxytricha*, the DNA cleavage during genome rearrangement is carried out by transposases from the telomere-bearing element (TBE) families, which are Tc1/mariner transposons encoding three open reading frames including a 42 kD transposase [91]. Analysis of the micronuclear genome found 10,109 complete TBEs, clustering into four families [3,92]. The transposons are expressed during conjugation and silencing through RNAi by feeding leads to failure to excise IESs and rearrange the genome [93]. This was shown by silencing the transposases using twelve different RNAi silencing constructs corresponding to a number of different identified versions of the TBE transposase genes, meaning that because of high sequence similarity, a large number of transposases would be silenced. It is not known what proportion of the transposases is required for genome rearrangement, but importantly silencing of all four families gave a much stronger effect than individual silencings [93]. It seems plausible that a large number if not all of the functional transposases are involved in the genome rearrangement process. This indicates that in *Oxytricha*, rather than domesticating a single or few transposases and upregulating them during development like *Paramecium* and *Tetrahymena*, the cells relies on the germline-limited expression of thousands of complete transposable elements, which mediate their own removal from the germline along with that of other IESs and unwanted DNA. This is a good example of a mutually beneficial relationship between parasitic DNA and its host [94].

The global expression of thousands of transposable elements raises the question of why this does not lead to reintegration of further copies of transposons into the germline at each sexual cycle. The answer to this is not clear, but may be related to the rapid circularisation of excised TEs and IESs.

## Summary

As has been shown, the developmental processes of ciliates have many broad similarities, although they differ in the mechanistic details of how they remove noncoding and repetitive DNA from their somatic genomes. The similarities include transposon domestication and/or mutualism, the importance of Piwi-associated small RNAs, and the use of the old maternal macronucleus as a template for the formation of the new zygotic macronucleus. The latter makes ciliates a fascinating model for the study of epigenetic inheritance of acquired characteristics, as the maternal macronucleus is somewhat plastic over vegetative divisions [95]. The mechanistic differences in the pathways between different ciliates often reflects their different structures and requirements. For example, the piRNAs in *Oxytricha* target sequences for retention rather than elimination, reflecting the relatively small proportion of sequences retained in *Oxytricha* (~5%) compared with *Paramecium* and *Tetrahymena* (~75% and 65%, respectively). Even in the more closely related species *Tetrahymena* and *Paramecium*, mechanistic differences reflect differences in IES localisation and size. For example, late-scnRNAs in *Tetrahymena* can be directly transcribed from unexcised IESs, whereas in *Paramecium,* the short IES size means that to avoid excessive MDS transcription, it is more efficient to concatenate already excised short IESs and transcribe them into iesRNAs.

Importantly, at every step in the genome rearrangement pathways, noncoding RNAs are crucial, and often have homologous functions and binding proteins even in widely divergent ciliate species. This makes ciliates a useful model organism for discovering novel uses for RNA, and demonstrates the importance of noncoding RNA in ancient eukaryotes.

## Funding

This work was supported by grants from the 10.13039/501100000781European Research Council (ERC) (681178 “G-EDIT”), the 10.13039/501100001711Swiss National Science Foundation (31003A_146257 and 31003A_166407), and from the 10.13039/501100009149National Center of Competence in Research (NCCR) RNA and Disease, Switzerland.
